# Greater lean tissue and skeletal muscle mass are associated with higher bone mineral content in children

**DOI:** 10.1186/1743-7075-7-41

**Published:** 2010-05-11

**Authors:** Karen B Dorsey, John C Thornton, Steven B Heymsfield, Dympna Gallagher

**Affiliations:** 1 Department of Pediatrics, Yale University School of Medicine, New Haven, CT, USA; 2 The New York Obesity Research Center, St Luke's-Roosevelt Hospital Center, College of Physicians and Surgeons, Columbia University, New York, NY, USA; 3 Merck, Inc, Rahway, NJ, USA; 4 The Institute of Human Nutrition, Columbia University, New York, NY, USA

## Abstract

**Background:**

To compare the relationship of skeletal muscle mass with bone mineral content in an ethnically diverse group of 6 to 18 year old boys and girls.

**Methods:**

175 healthy children (103 boys; 72 girls) had assessments of body mass, height, and Tanner stage. Whole body bone mineral content, non-bone lean body mass (nbLBM), skeletal muscle mass, and fat mass were assessed using dual-energy X-ray absorptiometry (DXA). Muscle mass was estimated from an equation using appendicular lean soft tissue measured by DXA, weight and height. Estimates of skeletal muscle mass and adipose tissue were also assessed by whole body multi-slice magnetic resonance imaging (MRI). Linear regression was used to determine whether skeletal muscle mass assessed by DXA or by MRI were better predictors of bone mineral content compared with nbLBM after adjusting for sex, age, race or ethnicity, and Tanner stage.

**Results:**

Greater skeletal muscle mass was associated with greater bone mineral content (p < 0.001). The skeletal muscle mass assessed by MRI provided a better fitting regression model (determined by R^2 ^statistic) compared with assessment by DXA for predicting bone mineral content. The proportion of skeletal muscle mass in nbLBM was significantly associated with greater bone mineral content adjusted for total nbLBM.

**Conclusions:**

This study is among the first to describe and compare the relationship of skeletal muscle to bone using both MRI and DXA estimates. The results demonstrate that the use of MRI provides a modestly better fitting model for the relationship of skeletal muscle to bone compared with DXA. Skeletal muscle had an impact on bone mineral content independent of total non-bone lean body mass. In addition, Hispanics had greater bone mineral content compared to other race and ethnic groups after adjusting for sex, age, adipose tissue, skeletal muscle mass, and height.

## Background

Fat-free body mass as measured by dual X-ray absorptiometry (DXA) consists of 50% bone and extracellular fluids, and 50% non-bone lean body mass (nbLBM) including muscle, organs, and connective tissue[1]. In a 1992 review of human studies, Weinsier et al. reported that as children grow bone is consistently 50% of fat-free body mass. However, muscle becomes an increasingly greater component. For example, Weinsier et al reported that fat-free mass was comprised of 30% muscle in infants and pre-school aged children, and 44% muscle in adults. Thus, the ratio of skeletal muscle mass to fat free body mass is not constant during growth. Consequently, estimates of fat-free body mass or of nbLBM might not accurately reflect the relationship of skeletal muscle mass to bone in children.

In 2006, Kim et al. described a new method for estimating skeletal muscle mass from whole body DXA scans and compared these estimates with skeletal muscle mass measured by whole body magnetic resonance imaging[2]. The assessment of skeletal muscle mass from DXA or MRI allows for the isolation of skeletal muscle mass from total nbLBM in the limbs and whole body respectively. These new techniques make it possible to determine how changes in skeletal muscle mass as a component of nbLBM relate to changes in bone mineral content in children[3].

Several studies examining the association between nbLBM and bone mineral content, using DXA, have demonstrated that for individuals of the same total body mass, nbLBM has consistently been a predictor of bone mineral content[4-7]. Studies have also demonstrated an association between muscle strength and bone area in children using computed tomography of individual limbs (typically the arm)[6,8-10]. In addition, a study by Wang et al. found that skeletal muscle measured by total body potassium in children was significantly associated with bone mass measured by total body calcium[11]. Results from these studies suggest that the relationship between skeletal muscle and bone mineral content in children is affected by sex, pubertal development, and race or ethnicity. Until recently however, it has not been possible to clarify these relationships using whole body estimates of skeletal muscle mass.

The purpose of this study was to directly compare DXA-based measures of nbLBM, DXA-based estimates of skeletal muscle mass, and MRI-based measures of skeletal muscle mass with respect to their relationship to bone mineral content in an ethnically diverse sample of 6 to 18 year old boys and girls. The relationship between bone mineral content and the ratio of skeletal muscle mass to total nbLBM was also determined. The influence of age, sex, race or ethnicity, adipose tissue (by MRI), and pubertal development on these relationships was studied.

## Methods

Subjects were 175 children ages 6 to 18 years of age who participated in two separate studies. One study recruited subjects between the ages of 6 to 18 years of age (n = 142). The second study recruited subjects from 7 to 11 years of age (n = 33). For both studies, all data were collected at the New York Obesity Research Center at St. Luke's Roosevelt Hospital. All subjects were recruited through schools, local area newspaper advertisements, and flyers posted in various locations in the local community. Children were eligible to enroll if they were healthy (had no chronic medical conditions), had a body mass index (BMI) less than 35 kg/m^2 ^(for adolescent subjects) and were not taking medications that could affect appetite, metabolism, or growth. Written consent was obtained from parents of all participating children. In addition, verbal assent was obtained from all children and written assent from children older than 7 years of age. Approval was obtained from the Institutional Review Board at the St. Luke's Roosevelt Hospital before enrollment and data collection. All appropriate protections for human subjects were followed in the study procedures data management and reporting of results.

Demographic and anthropomorphic measures were collected on each subject, body mass measured by a digital scale (Avery Weigh-Tronix digital scale, model DS-01, Pointe-Claire, Quebec, Canada) to the nearest 0.1 kg and height measured with a wall mounted digital stadiometer (235 Heightronic Digital Stadiometer, Quick Medical and Measurement Concepts, Snoqualmie, WA) to the nearest 0.1 cm. A medical exam was performed by a pediatrician after each subject's enrollment to assess: 1) Tanner stage according to breast and pubic hair development in girls and testicular and pubic hair development in boys; and, 2) overall physical health. Tanner stage (n = 122) was simplified to Tanner category (1 = pre-pubertal, 2 to 4 = pubertal, 5 = late puberty) in these analyses since stages 2 to 4 have been most associated with rapid growth of bone and muscle tissues compared with stages 1 and 5 where growth is more modest[12,13]. Race and ethnicity were assessed by questionnaire reporting of the race or ethnic background of the participating child (the possible categories were Asian, Black, White, Hispanic, and other).

**Figure 1:**
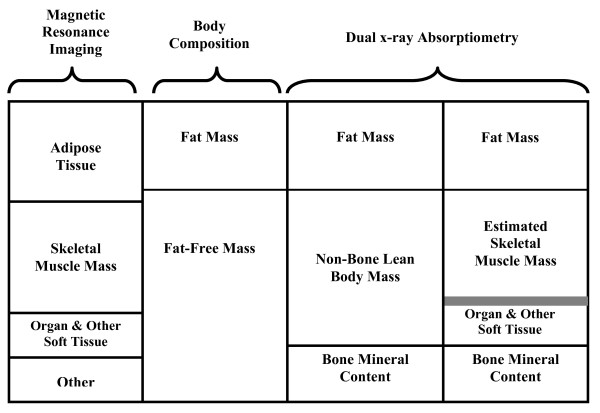
**Body Composition Components Estimated by Dual x-ray Absorptiometry and Magnetic Resonance Imaging**. There is a shaded gray barrier between the estimated skeletal muscle mass block and the organ and other soft tissue block in the 4th column under "dual x-ray absorptiometry" estimates. This conveys the SMM_DXA _is an estimate of total body musculature based on limb muscle mass only. Thus there is likely to be variability in the accuracy of SMM_DXA _values for individual subjects with respect to the estimated amount of axial musculature (e.g., for some it may be overestimated, for others it may be underestimated).

Whole body DXA was used to assess bone mineral content in all subjects using the Lunar Prodigy (GE Medical, Madison, WI). Four software versions were used between 2003 and 2006 including versions 6.7, 6.8, 8.1, 8.8. Each scan provided estimates of total fat-free mass, total and regional bone mineral content, non-bone lean body mass (nbLBM), and fat mass (Figure 1). In our laboratory the coefficients of variation were 5.3% for nbLBM and 9.9% for fat mass measurements on the Lunar Prodigy (based on monthly values for water and methanol phantom assessments from January 2003 through December 2006). Appendicular lean soft tissue (ALST) was the sum of non-bone lean body mass (total fat free mass - total bone mineral content) in the right and left legs and arms and was calculated using computer generated and manually confirmed default lines on anterior view planogram as previously described[2,14,15]. Default lines are based on anatomic landmarks including the perpendicular axis of the femoral neck angled with the pelvic brim to the tips of the phalanges in the legs and the center of the arm socket to the phalange tips in the arms. Estimates of total body skeletal muscle mass (SMM_DXA_) were calculated from ALST values, height, and weight for each subject using a prediction equation developed for pediatric samples by Kim et al[2].(1)

Although SMM_DXA _values are estimates of total body muscle mass they are derived from limb musculature only. Thus SMM_DXA _values might be less accurate than a direct measure of total body muscle from MRI. This difference in the accuracy of SMM_DXA _and SMM_MRI _might affect their relationships to bone mineral density. The current analysis included subjects from one study who contributed data to the prediction equations by Kim et al. (n = 142) as well as subjects from a second study (n = 33). Thus, this analysis does not represent an external validation of the prediction equation developed by Kim et al.

Whole body multi-slice MRI was used to determine skeletal muscle mass (SMM_MRI_) for all subjects. A 1.5-T scanner was used (6X Horizon: GE Milwaukee, WI) and images created with T-weighted spin-echo sequence and a 210 ms repetition time and echo time of 17 ms. Subjects lay motionless on the scanner platform with arms extended above their heads. The origin of all scans was set at the inter-vertebral space between L4 and L5. Transverse images were then acquired for the whole body with a between slice gap of 40 mm in taller pediatric subjects and 25 or 35 mm gaps for smaller pediatric subjects generating between 30 and 40 image slices per subject[2,16].

The SLICEOMATIC (TomoVision Inc, Montreal, Canada) software was used to calculate cross sectional tissue area and segmentation of tissue compartments including separation of intra-muscular adipose tissue from skeletal muscle according to previously established protocols [17]. Total body skeletal muscle and adipose tissue volumes were converted to mass using the estimated density of both tissues (1.04 kg/L for muscle and 0.92 kg/L for adipose tissue)[18]. The intra-class correlation coefficient for MRI estimates of skeletal muscle mass has been reported as 0.99 in adults [19]. In addition, technical error for repeated estimates of skeletal muscle mass in adults from whole body MRI imaging with a single trained analyst is 1.4% in our laboratory[19].

Figure 1 depicts the elements of fat mass and fat-free mass identified by MRI and DXA respectively. Elements of body tissues are identified from MRI by marking the physical boundaries of tissues visualized in each scan (fat, muscle, and organs), measuring their volume, and estimating their mass. Body tissues are identified from DXA by determining the absorption of x-ray beam according to differences in tissue properties (i.e. differences in the absorption of bone compared with muscle or fat). Thus, adipose tissue measured by MRI contains fat mass as well as the connective tissue that surrounds fat cells, and tends to be larger than DXA based estimates of fat mass alone. In addition, skeletal muscle is directly visualized in the trunk and limbs and can therefore be quantified by whole body MRI. Although the distinct absorption of x-ray beams from DXA scans allows for measurement of nbLBM, skeletal muscle mass must then be estimated from these values (e.g. using the equation by Kim et al.).

### Statistical Methods

**Figure 2:**
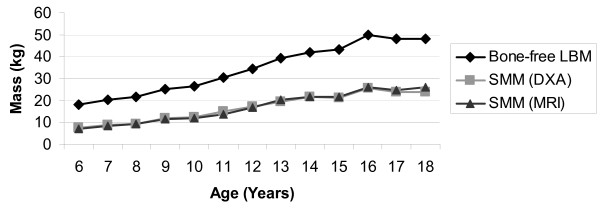
**Skeletal Muscle Mass by DXA and MRI, and Total Non-Bone Lean Body Mass as a Function of Age (years)**.

Descriptive statistics were calculated for demographic and body composition variables using means and standard deviations (SD) for dimensional variables and frequencies for categorical variables. In order to examine how well the Kim et al. prediction equation approximated MRI estimates of skeletal muscle mass, we examined body composition variables for subjects who participated in the Kim et al. validation study (n = 142) and those who participated in the second study (n = 33) separately. Mean estimates of non-bone lean body mass, and skeletal muscle mass from DXA and MRI (SMM_DXA_, and SMM_MRI_) were calculated for male and female children at each sample and plotted as a function of age (Figure 2). Subjects from both studies were combined for the subsequent analyses.

Multiple linear regression was used to develop a model to determine whether skeletal muscle mass was independently associated with greater bone mineral content adjusting for the variables found to be significantly associated with either variable including age, sex, race or ethnicity, Tanner stage, and adipose tissue. Multiple linear regression was also used to describe the relationship of the proportion of nbLBM comprised of skeletal muscle mass (SMM_MRI_•nbLBM^-1^) to bone mineral content controlling for total nbLBM, and other independent variables (sex, age, height, adipose tissue, race/ethnicity). The hypothesis that skeletal muscle mass from MRI (SMM_MRI_) would be a better predictor of bone mineral content (BMC) compared with skeletal muscle mass from DXA (SMM_DXA_) and nbLBM was tested using linear regression. The different estimates for skeletal muscle mass and nbLBM (SM_MRI_, SMM_DXA_, and nbLBM) were compared with respect to their association with bone mineral content using the semi-partial correlation coefficient in multiple linear regression models. Dummy variables were assigned for sex (s = 1 for boys and s = 0 for girls), pubertal stage (for Tanner stage 1, t1 = 1 for pre-pubertal and t1 = 0 for all others; for Tanner stages 2 to 4, t2 = 1 for pubertal and t2 = 0 for all others), race or ethnicity (for Black subjects, b = 1 and b = 0 for all others; Hispanic, l = 1 and l = 0 for all others; Asian, a = 1 and a = 0 for all others; and, White, w = 1 and w = 0 for all others). Tanner stage data were available for only a subset of 129 subjects. Although pre-pubertal children were found to have lower adipose tissue and bone mineral content compared with pubertal children, the R^2 ^value of multivariate models predicting bone mineral content only improved by 0.002 when Tanner stage was included (other covariates included age, sex, skeletal muscle mass, adipose tissue, and race/ethnicity). Thus, Tanner stage was not included in the analyses. Linearity of relationships between dependent and independent variables were assessed using scatter plots and residual plots to explore relationships between independent and dependent variables; and transformations were studied including interactions by testing products among variables. Logarithmic conversion of skeletal muscle mass, bone mineral content, nbLBM and adipose tissue improved the linearity of regression models and was therefore used in the models presented. All statistical analyses were performed using the SAS version 9.1 software package (SAS Institute INC, Cary, NC) for personal computer. The level of significance for all statistical tests of hypotheses was 0.05.

## Results

Similar trends in sexual dimorphism were observed in the original validation and external samples (Table 1). The values for skeletal muscle mass (SMM_DXA _estimated using the prediction equation, Kim et al., and SMM_MRI_) were similar for boys and girls in both the subjects used in the Kim et al. study and subjects from the second study. As expected, estimates of non-bone lean body mass (nbLBM) were consistently higher compared with estimates of skeletal muscle mass (SMM_DXA _and SMM_MRI_) as nbLBM contains organ and non-muscle soft tissue in addition to muscle (Figure 2). The absolute difference between nbLBM and estimates of skeletal muscle (SMM_MRI_) became greater with increasing age (increased from 10.8 kg among 6 to 8 year-olds to 19.1 kg among 13 to18 year-olds). However, the proportion of nbLBM comprised of skeletal muscle also increased from approximately 39-42% among 6 to 8 year old children to 50-52% among 16 to 18 year old adolescents.

**Table 1:** Demographic and Anthropometric Characteristics*

	Subjects from Study 1		Subjects from Study 2	
	
	6 to 18 years		7 to 11 years	
	
	Male (n = 83)	Female (n = 59)	Male (n = 20)	Female (n = 13)
**Age (yr)**	12.0 ± 3.7	11.6 ± 3.6	8.8 ± 1.3	8.2 ± 1.0

**Height (cm)**	154.1 ± 19.9	149.1 ± 14.0	136.2 ± 8.7	129.1 ± 8.4^**†**^

**Weight (kg)**	51.5 ± 21.2	47.7 ± 17.6	34.5 ± 10.0	30.1 ± 10.4

**FM_DXA _+ FFM_DXA_**	51.1 ± 21.4	46.9 ± 17.6	33.7 ± 9.9	29.3 ± 10.4

**BMI Percentile**	67.5 ± 26.7	66.4 ± 28.8	64.7 ± 32.0	65.7 ± 22.2

**Race/Ethnicity: n (%)**				
**White**	12 (14)	8 (13)	4 (20)	1 (8)
**Black**	31 (37)	27 (46)	5 (25)	5 (38)
**Hispanic**	28 (34)	20 (34)	7 (35)	1 (8)
**Asian**	3 (4)	1 (2)	4 (20)	6 (46)
**Other**	9 (11)	3 (5)	0	0

**FM_DXA _(kg)**	11.8 ± 9.3	15.3 ± 10.1^**†**^	8.8 ± 6.3	8.5 ± 5.8

**AT_MRI _(kg)**	12.4 ± 8.2	15.3 ± 9.3	10.0 ± 5.8	9.0 ± 5.6

**BMC_DXA _(kg)**	2.0 ± 0.9	1.8 ± 0.6	1.2 ± 0.3	1.0 ± 0.3^**†**^

**FFM_DXA _(kg)**	39.3 ± 15.3	31.7 ± 8.9^**†**^	24.9 ± 4.5	20.8 ± 4.9^**†**^

nb**LBM_DXA _(kg)**	37.3 ± 14.4	29.9 ± 8.3^**†**^	23.7 ± 4.3	19.8 ± 4.7^**†**^

**SMM_DXA _(kg)**	18.3 ± 8.4	14.3 ± 4.8^**†**^	10.4 ± 2.7	8.6 ± 2.4

**SMM_MRI _(kg)**	18.3 ± 8.9	14.4 ± 5.1^**†**^	9.9 ± 2.7	8.3 ± 2.8

*Subjects from Study 1 are those whose data were used to develop the prediction equations by Kim et al. All values are Mean ± SD. nbLBM_DXA _is total non-bone lean body mass measured by dual x-ray absorptiometry (DXA), SMM_DXA _is skeletal muscle mass estimated using DXA derived non-bone appendicular lean soft tissue (from the arms and legs) using the pediatric prediction equation developed by Kim et al., 2006, and SMM_MRI _is skeletal muscle mass measured by magnetic resonance imaging. Bone mineral content (BMC_DXA_), Fat Mass (FM_DXA_), and Fat-Free Mass (FFM_DXA_) are measured by DXA. Adipose tissue (AT) is measured by MRI scan. BMI is the body mass index (weight in kg/height in m^2^) ^**†**^Significant differences between boys and girls were found in the two study samples (p < 0.05).

Estimates of skeletal muscle based on MRI (SMM_MRI_) provided the best fitting model for predicting bone mineral content (BMC) using simple linear regression followed by nbLBM (SMM_MRI _R^2 ^= 0.948, β = 0.90, p < 0.001; nbLBM R^2 ^= 0.936, β = 1.16, p < 0.001). The estimate of skeletal muscle mass by DXA (SMM_DXA_) was the weakest predictor of bone mineral content (SMM_DXA _R^2 ^= 0.929, β = 0.94, p < 0.001) (Table 2). In multiple regression models, being male, increasing age, greater adipose tissue (AT), greater muscle mass by MRI, Hispanic ethnicity, and greater height were associated with greater bone mineral content (Table 3). There was a significant interaction between sex and adipose tissue (AT) which indicates the effect of adipose tissue (AT) varies by gender. The coefficients are 0.117 (SE = 0.019, p < 0.001) for girls and 0.030 (0.017, p = 0.082) for boys.

**Table 2:** Simple Regression Analysis Predicting the Logarithm of Bone Mineral Content [Log(BMC)] from the Logarithms of Non-bone Lean Body Mass, Skeletal Muscle Mass from DXA, and Skeletal Muscle Mass from MRI [Log(nbLBM), Log(SMM _DXA_) and Log(SMM_MRI_)]*

	MRI	DXA	
	
	Log(SMM_MRI_)	Log(nbLBM)^†^	Log(SMM_DXA_)
**R^2^**	0.948	0.936	0.929
**Parameter Estimate**	0.901	1.163	0.940

*Estimates of the natural log of non-bone lean body mass (nbLBM) and of skeletal muscle were significantly associated with the natural log of BMC in all models (p < .001). ^†^Total nbLBM was estimated from whole body DXA scans. Skeletal muscle mass was estimated by using measures of non-bone appendicular lean soft tissue (from the arms and legs) from DXA scans in the pediatric prediction equation developed by Kim et al., 2006 (SMM_DXA_).

**Table 3:** Predicting the Logarithm of Bone Mineral Content [Log(BMC)] from the Logarithm of Skeletal Muscle Mass from MRI [Log(SMM_MRI_)] Adjusting for Sex, Age, the Logarithm of Adipose Tissue [Log(AT_MRI_)], Ethnicity, and Height.

	β	SE_β_	SPCC^2^_β_†	P_β_	Model R^2^	SE	p model*
**Sex**	0.185	0.052	0.002	<0.001			
**Age**	0.008	0.004	0.001	= 0.033			
**Log(AT_MRI_)**	0.117	0.019	0.007	<0.001			
**Log(SMM_MRI_)**	0.542	0.046	0.024	<0.001			
Hispanic*	0.038	0.013	0.002	= 0.004			
**Height**	0.007	0.001	0.006	<0.001			
Interaction (sex*log(AT_MRI_))	-0.087	0.021	0.003	<0.001			

**Model Intercept**	-2.410	0.096		<0.001	0.970	0.079	<.0001

* The statistical significance of each variable in the model is indicated in the column headed "p". The model coefficient for each variable and the model intercept is identified as β and the standard error of each coefficient as SE_β_. The abbreviation "AT_MRI_" refers to adipose tissue estimated by whole body magnetic resonance imaging (MRI). † The squared semi-partial correlation coefficient (SPCC^2^) represents the total variation in the dependent variable after the independent variable is controlled for all other variables in the model. It was estimated in SAS (Cary, NC) for each independent variable in the model using the "scorr2" command.

The skeletal muscle proportion of nbLBM (SMM_MRI•_nbLBM^-1^) was associated with greater bone mineral content (β = 0.986, SE_β _= 0.247, p < 0.001) independent of the impact of total nbLBM (β = 0.607, SE_β _= 0.081, p < 0.001) (Table 4**)**. Multiple regression analysis showed that being male, greater age, greater adipose tissue, greater nbLBM, Hispanic ethnicity, and greater height were all significant predictors of greater bone mineral content when the skeletal muscle proportion of nbLBM was included in the model (for all variables p ≤ 0.05; Table 4).

**Table 4:** Predicting the Logarithm of Bone Mineral Content [Log(BMC)] from the Skeletal Muscle Proportion of Non-Bone Lean Body Mass (SMM_MRI _• nbLBM^-1^) Adjusting for Sex, Age, the Logarithms of Adipose Tissue and Non-Bone Lean Body Mass [Log(AT_MRI_) and Log(nbLBM), Ethnicity, and Height.

	β	SE_β_	SPCC^2^_β_†	P_β_	Model R^2^	SE	p model*
**Sex**	0.174	0.053	0.002	<0.001			
**Age**	0.007	0.004	0.001	= 0.050			
**SMM_MRI _• nbLBM^-1^**	0.986	0.247	0.003	<0.001			
**Log(AT_MRI_)**	0.121	0.019	0.007	<0.001			
**Log(nbLBM)**	0.607	0.081	0.010	<0.001			
Hispanic*	0.039	0.013	0.002	= 0.004			
**Height (cm)**	0.007	0.001	0.004	<0.001			
Interaction (sex*Log(AT_MRI_))	-0.086	0.021	0.003	<0.001			

**Model Intercept**	-3.401	0.092		<0.001	0.971	0.078	<.0001

* The statistical significance of each variable in the model is indicated in the column headed "p". The model coefficient for each variable and the model intercept is identified as β and the standard error of each coefficient as SE_β_. ^† ^The squared semi-partial correlation coefficient (SPCC^2^) represents the total variation in the dependent variable after the independent variable is controlled for all other variables in the model. It was estimated in SAS (Cary, NC) for each independent variable in the model using the "scorr2" command.

Substituting the DXA estimates of skeletal muscle mass and nbLBM in the multiple linear regression models (from Tables 3 and 4) produced results similar to those with MRI estimates of skeletal muscle mass (R^2 ^= .966, SE = .084 {Model 3 in Table 5}, R^2 ^= .967, SE = .081{Model 6 in Table 5}, and R^2 ^= .969, SE = .080{Model 2 in Table 5}). However, the MRI estimate of skeletal muscle mass accounted for the same amount of variance in the model predicting bone mineral content (using the square of the semi-partial correlation coefficient SPCC^2 ^= 0.024) compared with nbLBM (SPCC^2 ^= 0.024) and more variance compared with the DXA estimate of skeletal muscle mass (SPCC^2 ^= 0.021, Table 5). The variance attributable to SMM_DXA_**• **nbLBM^-1 ^was similar to that for SMM_MRI_**• **nbLBM^-1 ^(β = 0.986, SE_β _= 0.247, SPCC^2 ^= 0.003 for SMM_MRI_**• **nbLBM^-1 ^and β = 0.198, SE_β _= 0.093, SPCC^2 ^= 0.001 for SMM_DXA_**• **nbLBM^-1^, Table 5).

**Table 5:** Comparison of predictors of the Logarithm of Bone Mineral Content [Log(BMC)]: Log(SMM _DXA_), Log(SMM_MRI_), and SMM_MRI _• nbLBM^-1^

Variables for Fat Mass and Either Lean Body Mass or Skeletal Muscle Mass Included in the Model*	β	SE_β_	SPCC^2^_β_†	P_β_	Model R^2^	SE	p model
**Model 1:**					0.970	0.079	<.0001
**AT_MRI_**	0.117	0.019	0.007	<0.001			
**SMM_MRI_**	0.542	0.046	0.024	<0.001			

**Model 2:**					0.968	0.080	<0.0001
**FM_DXA_**	0.124	0.015	0.013	<0.001			
**nbLBM**	0.796	0.070	0.024	<0.001			

**Model 3:**					0.966	0.084	<0.0001
**FM_DXA_**	0.101	0.016	0.008	<0.001			
**SMM_DXA_**	0.530	0.053	0.021	<0.001			

**Model 4**					0.960	0.092	<0.0001
**AT_MRI_**	0.125	0.022	0.008	<0.001			
**SMM_MRI_•nbLBM^-1^**	0.839	0.111	0.014	<0.001			

**Model 5:**					0.971	0.078	<0.0001
**AT_MRI_**	0.121	0.019	0.007	<0.001			
**SMM_MRI_•nbLBM^-1^**	0.986	0.247	0.003	<0.001			
**nbLBM**	0.607	0.081	0.010	<0.001			

**Model 6:**					0.969	0.081	<0.0001
**AT_MRI_**	0.133	0.020	0.009	<0.001			
**SMM_DXA_•nbLBM^-1^**	0.198	0.093	0.001	0.035			
**nbLBM**	0.746	0.072	0.020	<0.001			

*All models are adjusted for age, gender, height, Latino ethnicity, and the interaction between fat mass and gender. ^**† **^The squared semi-partial correlation coefficient (SPCC^2^) represents the total variation in the dependent variable after the independent variable is controlled for all other variables in the model. It was estimated in SAS (Cary, NC) for each independent variable in the model using the "scorr2" command.

## Discussion

This study is one of the first to examine the independent impact of skeletal muscle mass alone on bone mineral content or to compare various estimates of skeletal muscle mass with respect to the relationship with bone mineral content in children. There was overlap among the estimates of nbLBM and skeletal muscle mass. When all three estimates of skeletal muscle mass and non-bone lean body mass (nbLBM) were included in one model, only MRI estimates of skeletal muscle mass independently predicted bone mineral content. In addition, when each variable was considered in a model alone, the use of skeletal muscle mass measured by MRI produced the best fitting model predicting bone mineral content and accounted for a larger proportion of the variance in bone mineral content compared with the other measures. Skeletal muscle mass was a predictor of bone mineral content after adjusting for other subject characteristics such that for two individuals of the same gender, age, height, race/ethnicity, and adipose tissue mass, the individual with greater skeletal muscle mass had a higher bone mineral content. Our findings also suggest that the proportion of nbLBM comprised of skeletal muscle increased as a function of age. Inclusion of this proportion as well as total nbLBM provided the best fitting model predicting bone mineral content, such that for two individuals of the same gender, age, race/ethnicity, height, adipose tissue, and nbLBM, the one with the higher proportion of skeletal muscle relative to total nbLBM had a greater bone mineral content.

These findings suggest that skeletal muscle mass was the most important predictor of bone mineral content. Direct comparison of the linear regression model that included skeletal muscle mass (SMM_MRI_) alone (i.e., without nbLBM) with the model containing both the skeletal muscle proportion of nbLBM (SMM_MRI_• nbLBM^-1^) and total nbLBM shows that the R^2 ^values were similar. However, the amount of variance in bone mineral content attributable to SMM_MRI_• nbLBM^- ^^1 ^was small. This suggests that although each variable independently predicts bone mineral content, the inclusion of skeletal muscle proportion of nbLBM does little to improve the prediction of bone mineral content beyond the inclusion of the total nbLBM or skeletal muscle mass alone. The results also show that although the MRI estimate of skeletal muscle mass was the best predictor of bone mineral content, DXA estimates of nbLBM produced similar results, Therefore, either method could be used to estimate skeletal muscle mass and examine its relationship with other body tissues. The finding that the DXA estimate of skeletal muscle mass was the poorest predictor of bone mineral content suggests that there is little advantage in calculating this estimate rather than using DXA-derived nbLBM in children.

The increase in the contribution of skeletal muscle to total nbLBM as a function of age may have important implications for the relationship between body tissues and other aspects of physiology in growing children. For example, a study by Hsu et al., using whole body MRI, provided evidence that the increased contribution of skeletal muscle as a function of age corresponds to a decrease in the proportion of organ tissue in total nbLBM, specifically liver and brain[20]. In addition, this observed decrease in the proportional contribution of organ tissue explained, in part, the decline in resting energy expenditure in young adults compared with children. The mechanism underlying the association between the proportional content of skeletal muscle in nbLBM and bone mineral content has not been previously described. These findings, however, provide additional evidence that the proportional composition of total nbLBM is dynamic during growth, and that these changes have important physiologic implications. Specifically, this study suggests that the proportional contribution of skeletal muscle mass to total nbLBM is an independent predictor of bone mineral content in children. Thus, the use of nbLBM as a proxy for skeletal muscle mass in growing children might not accurately reflect the impact of skeletal muscle mass on bone.

Despite these findings, the positive association between skeletal muscle mass and bone mineral content observed in this study, as well as the relationships of other key variables with bone mineral content, was similar to the findings of other studies that relied on total nbLBM as the only measure of lean tissue [4-7,12]. For example, van Lagendonck et al. found that nbLBM measured by whole body DXA was associated with higher bone mineral density in a sample of 21 twin pairs of pre-menarchal 8-year old girls[6]. Higher fat mass was also associated with greater bone mineral density of the lumber spine. In a similar study of 778 two to 20-year old boys and girls, Ferreti et al. reported that total nbLBM was a stronger predictor of bone mineral content compared with other variables (using whole body DXA)[5]. In addition, Ackerman et al described the relationship between bone mineral content, fat mass, and nbLBM measured by DXA among 926 boys and girls ages 6 to 18 years[4]. They also reported that for individuals of the same weight, nbLBM was the strongest predictor of bone mineral content, and that fat mass was correlated with bone mineral content. This correlation between fat mass and bone mineral content only became negative after adjusting for total body weight. Given that fat free mass is a stronger predictor of bone, an increase in fat mass at a fixed body weight would predict a decrease in bone. Our models did not control for total body weight. Therefore, the association between fat mass and bone mineral content remained positive.

The impact of non-bone lean body mass on bone mineral content has been attributed in part to the influence of biomechanical usage on bone development[21]. According to this theory, bone development is influenced by mechanical forces (muscle force and bone length) and hormonal factors. The force that muscle exerts against bone is influenced by how much body mass the muscles and bones support, although some experts have speculated that the characteristics of muscles themselves such as strength and mass might also be important[9,10]. For example, there is some evidence that muscle strength (grip strength) is associated with bone cortical area (arm) measured by quantitative computer tomography in children[8,9]. These studies have been limited to the analysis of muscle and bone in individual limbs. In the current study, the proportion of nbLBM comprised of skeletal muscle mass increased as a function of age and was independently associated with bone mineral content. This suggests that attempts to describe the relationship of muscle and bone in growing children using nbLBM alone might not accurately reflect the true relationships. Measurement of whole body muscle may provide a better method for assessing these relationships.

In this study sex, age, and race or ethnicity influenced the relationships between skeletal muscle mass and bone mineral content in children and adolescents. Specifically, Hispanics had greater bone mineral content compared with non-Hispanics after adjusting for gender, age, adipose tissue mass, height, and skeletal muscle mass. It is important to note that this study was not powered to detect statistically significant differences between the various racial and ethnic groups represented in this study. These data were included to account for any potential influence of race or ethnicity on the relationships of primary interest (those between skeletal muscle mass and bone mineral content). Thus actual differences between racial and ethnic groups may exist that we were not able to detect. The study was conducted in New York City in a community with large populations of African-American and Latino families. The small number of Caucasian subjects relative the US population also limits our ability to generalize the results. Our findings, however, are consistent with studies that included large numbers of Caucasian subjects and were conducted in other geographical areas with respect to the relationships observed between muscle mass and bone mineral content. In addition, the over-representation of minority groups is an important aspect of our study as very few examinations of body composition or relationships between body tissues have included these groups. Our findings underscore the importance of examining the influence of race or ethnic difference on the relationships between bone mineral content and lean tissues in children. Specifically, the observed impact of race and ethnicity on bone mineral content detected in this study indicate that further studies that focus on Hispanic populations are warranted.

## Conclusions

Our findings suggest that the assessment of skeletal muscle from whole body DXA or MRI scans may be applied in future work to clarify the specific role of skeletal muscle mass, as a component of non-bone lean body mass, in the development of bone in growing children. The weaker association between DXA estimates of skeletal muscle mass with bone mineral content compared to both MRI estimates and non-bone lean body mass might be attributable to the inaccuracy of estimating total body skeletal muscle mass from limb muscle only. It is important to consider the small sample size of this study when interpreting these results, in particular the conclusions drawn regarding race or ethnic differences in the relationships reported. For example, the Asian children in our sample were Chinese-American and Korean-American according to their surnames and are therefore, not representative of all Asian populations[22]. Tanner staging information was unavailable on 26% of children in the sample. The latter limits our ability to interpret the observed influence of Tanner stage on the sex- and age-related variations in bone mineral content. In addition, there have been no studies describing the test-retest reliability of estimates of skeletal muscle mass using whole body MRI in children, although technical error is low and intra-class correlation coefficients high for adult subjects[19]. Despite these issues, this work suggests that the relationship between bone and muscle in childhood might be best understood by applying methods for estimating skeletal muscle mass alone, rather than relying on estimates of total non-bone lean body mass.

## Abbreviations

DXA: dual x-ray absorptiometry; MRI: magnetic resonance imaging; BMI: body mass index; SMM_DXA_: skeletal muscle mass by DXA; SMM_MRI_: skeletal muscle mass by MRI; SD: standard deviation; nbLBM: non-bone lean body mass; BMC: bone mineral content; β: the regression coefficient; SE_β_: the standard error for the regression coefficient; SPCC: semi-partial correlation coefficient.

## Competing interests

The authors declare that they have no competing interests.

## Authors' contributions

KBD developed the hypotheses in consultation with the other authors, completed the statistical analysis, and drafted the manuscript. JCT helped KBD refine the hypotheses tested and assisted with the statistical analysis of the data. SBH was the principal investigator for one of the studies providing the data for this analysis and helped KBD refine the hypotheses tested in this analysis. DG was the principal investigator for one of the studies providing data for this study, helped KBD refine the hypotheses tested in this analysis, and contributed to the design of the statistical analysis. All authors read, edited, and approved the final manuscript.
